# Reassessment of the Wing Feathers of *Archaeopteryx lithographica* Suggests No Robust Evidence for the Presence of Elongated Dorsal Wing Coverts

**DOI:** 10.1371/journal.pone.0093963

**Published:** 2014-04-07

**Authors:** Robert L. Nudds

**Affiliations:** Faculty of Life Sciences, University of Manchester, Manchester, United Kingdom; Monash University, Australia

## Abstract

Recently it was proposed that the primary feathers of *Archaeopteryx lithographica* (HMN1880) were overlaid by long covert feathers, and that a multilayered feathered wing was a feature of early fossils with feathered forelimbs. The proposed long covert feathers of *Archaeopteryx* were previously interpreted as dorsally displaced remiges or a second set of impressions made by the wing. The following study shows that the qualitative arguments forwarded in support of the elongated covert hypothesis are neither robust nor supported quantitatively. The idea that the extant bird wing with its single layer of overlapping primaries evolved from an earlier multilayered heavily coveted feathered forelimb as seen in *Anchiornis huxleyi* is reasonable. At this juncture, however, it is premature to conclude unequivocally that the wing of *Archaeopteryx* consisted of primary feathers overlaid with elongated coverts.

## Introduction

There are broadly two competing hypotheses (arboreal versus cursorial) for the evolution of flight in birds [1], [2]. Under these broad opposing umbrella ideas are further sub-theories that vary in their thoughts on the exact selection pressure driving that evolution [3], [4], [5], [6], [7], [8], [9]. Intrinsically linked to the evolution of flight is the evolution of the avian wing and central to this, is the interpretation of the morphology of the feathered wings of early fossil birds and their antecedents.

A recent paper by Longrich *et al*. [10] proposes a new primitive form of wing feather arrangement in early flying birds and hence a new scenario for the evolution of the avian wing. Specifically, early feathered forelimbs had multiple layers of wing covet feathers as seen in *Anchiornis* [11] and these gradually receded to become the single layered primaries seen in extant birds [10]. Central to the argument of Longrich *et al*. [10] is that the primary feathers of *Archaeopteryx lithographica* were overlaid by long covert feathers. These proposed coverts were previously interpreted as dorsally displaced remiges [12], [13] or a second set of impressions made by the wing [14]. Clearly, a wing constructed of multiple layers of flight feathers is different from the single overlapping primary feather arrangement seen in extant birds and has important implications for the interpretation of wing evolution and the flight capabilities of *Archaeopteryx*. For example, multiple layers of feathers would add strength to the wing surface and would mean that slots between the primary feathers could not be produced [10]. This extra strength would potentially add support to the primary feather rachises of *Archaeopteryx*, which are thin relative to body mass in comparison with extant birds [15], [16], [17], [18]. In contrast, the lack of ability to produce slots between the primary feathers would compromise aerodynamic performance, as slotting reduces induced drag during gliding and soaring flight [19], [20], [21]. Here, however, it is shown that the 3 pieces of evidence proposed in support of the obscured feathers of *Archaeopteryx* being coverts and not primary feathers [10] are not supported quantitatively and the qualitative arguments not robust.

## Materials and Methods

The angles of the feather rachises were calculated relative to the vertical in figures 1D and 2D of Longrich *et al*. [10]. A straight line was drawn from a point in the middle of the most proximal visible part of a rachis to the most distally visible part of the rachis as depicted either by solid grey lines (primaries) or dotted, and red lines (the proposed coverts). For balance, the left most covert in figure 1D [10] was not included in the analyses, because there is no corresponding primary feather impression. Inclusion of its angle, however, makes no difference to the results.

## Results and Discussion

The key piece of evidence forwarded for the presence of elongated wing covets on the Berlin specimen is that the obscured feather rachises are angled relative to the visible primary feathers [10]. This conclusion, however, is not supported quantitatively. There is no difference (Two-tailed test: *t* = 0.295, *p* = 0.776) between the angles of the primary feathers (16.54±0.69°) and proposed coverts (17.01±1.42°) on the left wing of *Archaeopteryx*. Neither is there a difference (Two-tailed test: *t* = 0.422, *p* = 0.688) between the angles of the primary feathers (57.09±0.62°) and proposed coverts (56.72±0.64°) on the right wing (Table 1), implying that they are in fact parallel.

**Table 1:** The calculated angles of the primary feathers and proposed coverts feather rachises on the left- and right-wing of *Archaeopteryx* (HMN1880).

Feather	Angle (°)
**Left wing**	
Proposed covert 2	21.95
Proposed covert 3	18.19
Proposed covert 4	15.88
Proposed covert 5	13.81
Proposed covert 6	15.23
Primary 1	19.15
Primary 2	15.61
Primary 3	15.20
Primary 4	16.20
Primary 5	16.57
**Right wing**	
Proposed covert 4	57.42
Proposed covert 3	55.53
Proposed covert 2	55.76
Proposed covert 1	58.17
Primary 5	58.84
Primary 4	56.96
Primary 3	55.97
Primary 2	56.61

Proposed coverts and primary feathers are numbered from left to right and bottom to top in figures 1D and 2D of Longrich *et al*. [10] respectively. The angles are calculated relative to the vertical axis in the figures.

Although differences in feather curvature were not suggested by Longrich *et al*. [10], the proposed covert between primaries 4 and 5 in their Figure 1D (left wing) appears to have no curvature, whereas primary 5 curves toward it. In addition, the proposed coverts between primaries 2 and 3, and 3 and 4 appear to be curving earlier than primaries 3 and 4 respectively, although the overall curvature appears similar (without visible rachis tips or calami curvature cannot be determined quantitatively). For the remaining feathers (both wings) the curvature of the proposed coverts appears to match that of the companion primary for each of the 6 pairs. Does 3 out of 9 pairs having different curvature or curving earlier constitute evidence for primary/covert pairs as opposed to just primaries? Primaries 5, 6 and 7 on each wing curve toward all more proximally positioned flight feathers including primaries 4–1. Therefore, it is not surprising that primary 5 also curves toward the proposed covert situated more proximally on the wing. That leaves 2 out of 9 proposed coverts that are curving earlier than their companion primaries, which could easily be the consequence of preservation, particularly as feathers are curved dorso-ventrally as well as medio-laterally, and these 2 axes of curvature are forced into 1 when flattened as fossils.

The second piece of evidence presented is that the tips of the obscured feathers are not visible at the wing margin and therefore, they are not as long as the prominent surface most primaries [10]. This is a flawed argument. Primary feathers narrow distally and in most cases only the rachis and not vane impressions are clearly visible in the proposed coverts. Because the proposed coverts are deeper in the slab and their impressions less prominent, it would be expected that the visibility of their vanes and rachises would peter out more distant from the wing margin than the more prominent impressions of the primaries overlaying them. It is simply a question of perspective.

The third argument put forward is that interpreting the obscured feathers as primaries requires that every other primary has been displaced post-mortem on both the left and right wings, without disturbance of the other primaries or ventral coverts and no taphonomic mechanism is known that could produce such a pattern [10]. Thus far nobody has run a taphonomic experiment that replicates the fossilisation process undergone by *Archaeopteryx* over 140My ago. Therefore, ‘no taphonomic mechanism currently known’ is not conclusive evidence of absence. Although speculative, it is possible that rather than the overlapping pattern seen in living birds (where the primaries are arranged with the trailing edge of each feather overlaying dorsally the leading edge of the adjacent), the primitive arrangement in *Archaeopteryx* was for the primaries to lay completely above or below, successively, the adjacent feathers when the wing was partially or completely folded. The anatomy of the *Archaeopteryx* manus and wrist, where the primary feathers attach, is certainly very different to that seen in extant birds [22], [23], [24], [25].

Therefore, contrary to the assertion of Longrich *et al*. [10], there is no robust evidence supporting a covert categorisation for the obscured feather impressions. They are just as likely to be primary feathers as earlier proposed [12], [13]. Hence, a reassessment of how the wing of *Archaeopteryx* functioned [10], for example, the wing not being capable of producing slots, or the coverts creating a second layer of wing-feathers strengthening thin rachised primary feathers [15], [16] (see also [18]) during flight, is at this point in time redundant. The idea that the general extant bird *bauplan* of a single, albeit overlapping, layer of primary feathers evolved from an earlier multilayered heavily coveted feathered forelimb as seen in *Anchiornis* [11] is appealing. The efficacy of a wing surface made out of multiple layers of small covets, however, is yet to be determined. Moreover, without more robust quantitative evidence, it is premature to conclude unequivocally that the primary feathers of *Archaeopteryx* were overlaid by elongated coverts.
